# Correspondence Between Cytomegalovirus Immunoglobulin-G Levels Measured in Saliva and Serum

**DOI:** 10.3389/fimmu.2020.02095

**Published:** 2020-08-28

**Authors:** Jenna L. Riis, Hedyeh Ahmadi, Olivia Silke, Steve W. Granger, Crystal I. Bryce, Douglas A. Granger

**Affiliations:** ^1^Institute for Interdisciplinary Salivary Bioscience Research, University of California, Irvine, Irvine, CA, United States; ^2^Department of Psychological Science, University of California, Irvine, Irvine, CA, United States; ^3^Salimetrics Research and Technology Center, Carlsbad, CA, United States; ^4^T. Denny Sanford School of Social and Family Dynamics, Arizona State University, Tempe, AZ, United States; ^5^Department of Acute and Chronic Care, Johns Hopkins University School of Nursing, Baltimore, MD, United States; ^6^Department of Pediatrics, Johns Hopkins University School of Medicine, Baltimore, MD, United States; ^7^Salivary Bioscience Laboratory, Department of Psychology, University of Nebraska–Lincoln, Lincoln, NE, United States

**Keywords:** cytomegalovirus, saliva, serum, immunoglobulin-G, antibody, cytokine

## Abstract

Human cytomegalovirus (HCMV) infects more than 80% of the global population. While mostly asymptomatic, HCMV infection can be serious among the immunocompromised, and it is implicated in chronic disease pathophysiology in adulthood. Large-scale minimally invasive HCMV screening could advance research and public health efforts to monitor infection prevalence and prevent or mitigate downstream risks associated with infection. We examine the utility of measuring HCMV immunoglobulin-G (IgG) levels in saliva as an index of serum levels. Matched serum and saliva samples from healthy adults (*N* = 98; 44% female; 51% white) were assayed for HCMV IgG, total salivary protein, and salivary markers related to oral inflammation, blood, and tissue integrity. We examine the serum-saliva association for HCMV IgG and assess the influence of participant characteristics and factors specific to the oral compartment (e.g., oral inflammation) on HCMV IgG levels and cross-specimen relations. We found a robust serum-saliva association for HCMV IgG with serum antibody levels accounting for >60% of the variance in salivary levels. This relation remained after adjusting for key demographic and oral immune-related variables. Compared to the serum test, the salivary HCMV IgG test had 51% sensitivity and 97% specificity. With improvements in assay performance and sample optimization, HCMV antibody levels in oral fluids may be a useful proxy for serum levels.

## Introduction

Cytomegalovirus (human betaherpesvirus 5, HHV-5, or HCMV) infection is ubiquitous in the human population, infecting individuals of all ages and approximately 83% of people worldwide (1). HCMV is transmitted primarily through bodily fluids (e.g., saliva, urine, and breast milk), and, even in healthy individuals, HCMV is not cleared by the immune system (2). HCMV can infect and replicate in most cell types throughout the body, and, after primary infection, HCMV establishes latency in early myeloid lineage cells (2, 3). Latent HCMV can become reactivated during periods of immunosuppression and/or re-exposure (4, 5). While most HCMV infections are asymptomatic, primary infection and viral reactivation can have serious negative health effects among the immunocompromised (e.g., HIV-infected individuals, organ transplant recipients) (3). Congenital infection, caused by maternal HCMV primary infection, reinfection, or reactivation during gestation, can also cause life-long health and developmental disorders including neurodevelopmental and cognitive problems and serious hearing loss (3, 6). In addition to the well-established health risks of HCMV among these special populations, in adults, HCMV has also been implicated in the pathophysiology of oral and systemic chronic diseases such as periodontal disease, cancer, cardiovascular disease, and neuropsychiatric disorders (7–12). Higher serum concentrations of HCMV antibodies have also been associated with indices of aging and immunosenescence, atherosclerosis, and a higher risk of all-cause mortality (7, 13–15). As with other herpes family viruses, elevated stress, via its association with immune suppression and decreased immune surveillance, may play a role in the negative health consequences of HCMV infection and the risk of chronic disease. HCMV replication and antibody levels have been found to increase during periods of stress and stress-related immunosuppression (16–20).

The impact of HCMV infection on health and development may be especially high among low-resource and racial/ethnic minority communities. Geographic, socioeconomic (SES), and racial/ethnic disparities in HCMV infection rates and prevalence worldwide are well documented with low SES and racial/ethnic minority groups disproportionately affected (21–23). The marked sociodemographic disparities in HCMV infections parallel disparities in the rates of exposure to adversity, and HCMV’s stress-linked mechanism for viral shedding and reactivation, raises the possibility that monitoring HCMV antibody levels could benefit public health efforts to prevent and mitigate downstream risks associated with infection (24). Serum HCMV immunoglobulin-G (IgG) tests are currently used in clinical settings (25). Significant associations between the reactivity and neutralizing capacity of serum IgG and salivary IgG (26, 27) suggest promising opportunities to measure HCMV prevalence using salivary IgG tests. Saliva-based HCMV testing platforms for immunoreactivity could help maximize the impact of HCMV screening by facilitating minimally invasive, community, field, and home-based testing for antibody levels on a large-scale. Such advances could support public health surveillance programs for HCMV and HCMV-related disorders and diseases, as well as expand clinical assessments for individuals at high risk of HCMV infection and/or infection complications (e.g., the immunocompromised and pregnant women).

The emerging field of salivary bioscience [see (28) for review], however, has cautioned that, when relating concentrations of analytes measured in oral fluids to systemic measures, oral-specific processes, such as local inflammation and tissue repair and reconstruction in the mouth, may confound their associations and ultimately limit their clinical utility (29, 30). Oral health and disease are therefore important potential confounders of salivary biomeasure concentrations that may compromise our ability to accurately monitor HCMV antibody levels in saliva. The potential impact of oral-specific confounds on salivary HCMV antibody screening is heightened among low SES and racial/ethnic minority communities where disparities in oral and physical health overlap with increased HCMV prevalence and infection rates (21, 22, 31, 32). Identifying sociodemographic and oral and physical health factors associated with HCMV antibody levels, and the influence of these factors on HCMV antibody serum-saliva associations, is an important step in developing a saliva-based HCMV testing protocol that could be widely and reliability implemented.

In the current investigation, matched serum and saliva samples from a study of healthy adults were examined to address these gaps in our understanding of salivary HCMV antibody levels. Specifically, we explore the distribution and demographic and health correlates of HCMV IgG levels in serum and saliva and examine the serum-saliva association for HCMV IgG levels. We evaluate the extent to which oral-specific confounds (i.e., salivary flow rate, and markers related to oral inflammation, tissue integrity, and blood leakage) are associated with salivary HCMV IgG levels and influence its serum-saliva relation. We further investigate whether the level of blood and the concentration of total IgG in saliva affect the nature and/or strength of the serum-saliva association for HCMV IgG. Finally, to assess the clinical relevance of salivary HCMV IgG levels, we examine the ability of the salivary HCMV IgG test results to accurately differentiate across HCMV serostatus subgroups.

We anticipate a significant positive serum-saliva association for HCMV IgG levels, and positive associations between salivary markers related to inflammation and tissue integrity and salivary HCMV IgG levels. We expect the serum-saliva relation for HCMV IgG is strengthened when these oral-specific confounds are accounted for in the models, and that the serum-saliva association for HCMV IgG increases with increasing salivary total IgG and with increasing blood leakage into saliva. We expect salivary HCMV IgG test results correspond with HCMV serostatus determinations.

## Materials and Methods

### Study Design, Sample, and Procedures

This study examines data collected from a cross-sectional study of healthy adults conducted in 2013–2014. Some of the data included in this study were examined in previous papers. The study design, participants, and methods, briefly summarized below, are the same as previously described [e.g., (30)].

A convenience sample was recruited via community postings. Potential participants were screened over the telephone, and those who were eligible were invited to participate in the study. During a single laboratory visit, study participants (*N* = 100) were asked to provide blood and whole saliva samples and complete a set of demographic and health questionnaires. Eligibility criteria excluded participants reporting chronic and/or acute health conditions, medication use (except hormonal contraceptives), open wounds or sores in their mouths, and recent oral surgery. In preparation for the study visit, participants were instructed to refrain from eating and drinking for at least 1 h prior to the visit. Study procedures were approved by the university’s Institutional Review Board, and participants were compensated $50.

#### Biospecimen Collection and Preparation

Resting samples of serum and saliva were collected from all participants. Whole blood was drawn by venipuncture into 2 mL lavender/EDTA tubes, and additional blood was drawn for serum isolation using an SST Tiger serum separator tube (BD #367988, Becton-Dickenson). Serum was mixed well by inversion and allowed to clot at room temperature for 30 min (and not longer than 1 h). After clot activation, serum tubes were spun in a refrigerated centrifuge at 2000 rpm for 10 min. Following centrifugation, serum was aliquoted into 2.0 mL Sarstedt cryovial tubes and frozen at −80°C until assay. Whole, unstimulated saliva was collected via passive drool. Saliva was mixed well by inversion, frozen to precipitate mucin, and then thawed to room temperature and mixed again by inversion and vortexing. Saliva was then centrifuged at 3500 rpm for 15 min, and the supernatant was transferred into 15 mL conical tubes. Following mixing by inversion and vortexing, saliva samples were aliquoted into 2.0 mL Sarstedt cryovials and stored at −80°C until assay. All serum and saliva assays were performed at the Johns Hopkins University Center for Interdisciplinary Salivary Bioscience Research laboratory.

### Measures

#### HCMV IgG

Salivary and serum HCMV IgG levels were assessed in duplicate using a diagnostic enzyme-linked immunosorbent assay designed for use in serum/plasma from IBL International (REF: RE57061). Serum samples were diluted 1:101 using 10 mM phosphate buffer pH 7.2 ± 0.2 and tested according to manufacturer’s protocol without modification. Saliva samples were assayed following the same protocol; however, to maximize IgG levels in the saliva, samples were tested without dilution (test volume = 100 μL). Salivary and serum HCMV IgG results were reported quantitatively [U/mL; (U = NovaTec Units)] and qualitatively using manufacturer-provided threshold values for a positive/negative test result. The cut-off value for HCMV IgG level was based on the assay’s “cut-off control” (absorbance value of 0.15–1.300; 10 NovaTec Units). Higher absorbance values indicated a positive test result, lower absorbance values indicated a negative test result, and samples within a 20% range of the cut-off control were considered equivocal. The intra-assay precision for salivary HCMV IgG tests was 5%, and the detection limit, determined for five sets of blank duplicates (substrate only), was 0.01 U/mL. The intra-assay coefficient of variation (CV) for serum tests was 5%.

#### Demographic and Health Characteristics

Demographic and health characteristics (Table 1) were examined as potential correlates of serum and salivary HCMV IgG levels and serostatus. Participants reported their age, sex, race, ethnicity, height, and weight on study questionnaires. Height and weight were used to calculate participant body mass index (BMI) (33). Participants also reported their self-perceived, current, physical health relative to other adults their age [Likert-type scale from 1 (excellent) to 5 (poor)]; their typical sleep quality [Likert-type scale from 1 (excellent) to 5 (poor)]; and the number of hours they typically sleep each night.

**TABLE 1:** Participant characteristics.

	*n* (%)
Age [mean years (*SD*)]	23.71 (4.56)
Female	41 (44.10)
**Race**	
White	44 (51.16)
African American	18 (20.93)
Other	24 (27.91)
**Ethnicity**	
Non-Hispanic	71 (88.75)
Hispanic	9 (11.25)
**Body mass index category**	
Underweight	4 (4.35)
Normal/Healthy weight	58 (63.04)
Overweight	25 (27.17)
Obese	5 (5.43)
**Current health**	
Excellent/Very good	68 (73.12)
Good	17 (18.28)
Fair/Poor	8 (8.60)
**General sleep quality**	
Excellent/Very good	45 (48.39)
Good	36 (38.71)
Fair/Poor	12 (12.90)
Typical hours of sleep per night [mean hours (*SD*)]	6.95 (1.08)
Oral Health Composite [mean score (*SD*)]	3.62 (0.84)

*All measures are based on self-reported data. Body mass index categories: underweight ≤ 18.50, normal/healthy weight = 18.5–24.9, overweight = 25.0–29.9, obese ≥ 30. Percentages are based on complete data per variable. Missing data = 4% for the oral health composite (n = 94); 5% for age, sex, current health, and general sleep quality (n’s = 93); 6% for body mass index and typical hours of sleep per night (n’s = 92); 12% for race (n = 86); 18% for ethnicity (n = 80). Oral health composite scores could range from 0 to 5, and higher scores represent better self-reported oral health.*

#### Oral-Specific Covariates and Confounders

The influence of factors related to the oral environment, including markers associated with oral health, inflammation, and salivary flow rate [see (30)], on salivary HCMV IgG levels and cross-specimen relations was examined using self-report and biologic indices.

Self-reported oral health was indexed by a series of five questions assessing participants’ frequency of brushing and flossing (per day and week, respectively), level of access to dental/oral health care [Likert-type scale from 1 (poor) to 5 (excellent)], and the presence of blood in saliva after oral care routines (yes/no) and of cuts/sores in the mouth (yes/no). Responses to these questions were rescaled and averaged to create a standardized self-reported oral health composite score [ranging from 0 to 5 with higher scores representing better self-reported oral health; see (29) for additional details].

Tissue remodeling in the oral compartment, a biologic marker related to oral health, was assessed with salivary matrix metalloproteinase-8 (MMP-8) concentrations (34). A commercially available development kit (DuoSet ELISA, R&D Systems, Cat# DY908) was used to assay salivary MMP-8 at a 1:50 dilution following the manufactures’ protocol. The assay has a range of sensitivity from 62.5 to 4000 pg/mL and the inter- and intra-assay CVs were 5 and 4%, respectively.

Oral inflammation was indexed by salivary concentrations of four proinflammatory cytokines [interleukin (IL)-1β, IL-6, IL-8, and tumor necrosis factor-α (TNFα)]. Cytokine concentrations were measured using a Meso Scale Discovery (MSD; Gaithersburg, MD, United States) 96-well format multiplex electrochemiluminescence immunoassay with a standard diluent (MSD# R51BB) following the manufacturer’s guidelines. The MSD Discovery Workbench Software (v. 3.0.17) determined cytokine concentrations using curve fit models (4-PL with a weighting function option of 1/y2). Lower limits of detection were <0.20 pg/mL, and the inter- and intra-assay CVs were less than 8 and 5%, respectively. Concentrations of the four cytokines were examined separately, and an average oral inflammation composite score was also created for each participant using standardized cytokine concentrations [*M* (SD) = 0.00 (0.75), range: −0.77– 3.34].

Blood in the oral compartment was indexed by concentrations of salivary transferrin (35, 36). Transferrin was measured using the Salimetrics enzyme immunoassay kit (State College, PA, United States; Cat# 1–1302) which has a range of sensitivity from 0.08 to 6.6 mg/dL. The inter- and intra-assay CVs were 5 and 3%, respectively.

Salivary total IgG was assayed following a laboratory-developed procedure using goat anti-human IgG [details provided in (29)]. Samples were diluted at 1:1250, and the calibration curve range ranged from 0.78 to 50 ng/mL. The average inter- and intra-assay CVs were <15% and <6%, respectively.

Salivary flow rate was indexed by concentrations of salivary total protein. Total protein was assessed using a Thermo Scientific assay kit (Pierce^TM^ BCA Cat# 23225). The assay was performed following the manufacturers’ guidelines.

### Statistical Approach

#### Analytic Sample, Model Diagnostics, and Sensitivity Analyses

One participant with incorrect serum HCMV IgG data was excluded from analysis, and two participants with missing serum HCMV IgG data were excluded from analyses examining serum IgG. Model fit and diagnostics were examined for all analyses. For each model, influential cases were identified, and sensitivity analyses assessed the robustness of the findings when cases with Cook’s Distance >1 and/or standardized residuals > ±3 were excluded. All linear regression models were performed using the full analytic sample, and sensitivity analyses were performed that stratified the sample by HCMV serostatus to assess whether the observed findings were driven by significant effects within a specific seronegative/seropositive subgroup. Analyses were conducted using Stata SE 15.1 (Stata Corp LLC, College Station, TX, United States) and R version 3.6.1 (R Core Team, 2019). Statistical significance was set at α = 0.05.

#### What Are the Distributions and Demographic and Health Correlates of HCMV IgG Levels in Serum and Saliva? Is Salivary HCMV IgG Level Correlated With Serum HCMV IgG Level?

**FIGURE 1:**
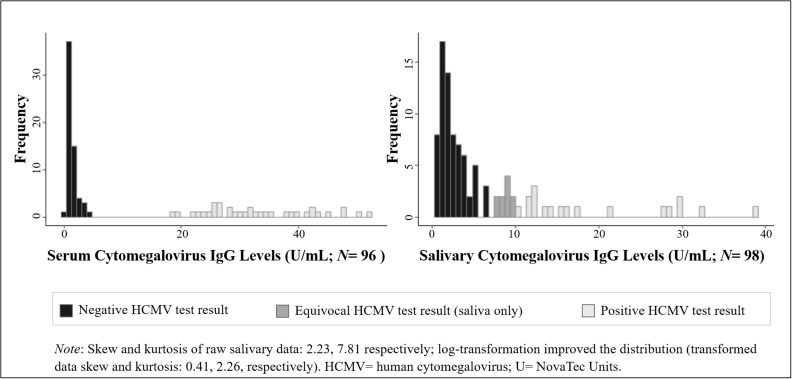
Distribution of human cytomegalovirus (HCMV) IgG levels measured in serum and saliva from healthy adults. (Data are coded to represent HCMV test results within biospecimen type).

We examined the distribution, range, and mean and median values of HCMV IgG in serum and saliva in our sample. We assessed the extent to which the demographic and physical health characteristics of our sample were associated with HCMV IgG levels and serostatus using parametric and non-parametric tests of association, as appropriate based on the distribution of the data. Variables significantly related to HCMV IgG levels and/or serostatus were included as potential covariates in subsequent models assessing the serum-saliva association for HCMV IgG. A non-parametric correlation (Kendall’s τ correlation) and a bivariate linear regression, assuming a log-normal distribution of salivary HCMV IgG (Figure 1), examined the unadjusted cross-specimen association for HCMV IgG levels. Differences in the serum-saliva association across serostatus groups were examined using an interaction between serum HCMV IgG level and serostatus in the linear regression model, and stratified correlation analyses were conducted to quantify the serum-saliva association within each serostatus group.

#### Are Salivary HCMV IgG Levels Associated With Oral Immune-Related and Saliva-Specific Confounds?

We used Kendall’s τ correlations to assess salivary HCMV IgG relations with the self-reported oral health composite score, and biologic indices related to oral inflammation (salivary cytokines), tissue integrity (salivary MMP-8), blood in saliva (salivary transferrin), and flow rate (salivary total protein).

#### Does the Relation Between Salivary and Serum HCMV IgG Levels Strengthen After Accounting for Oral Immune-Related Markers and Salivary Flow Rate? Does the Relation Between Salivary and Serum HCMV IgG Levels Increase With Increasing Levels of Blood and Total IgG in Saliva?

Multivariable linear regression models for salivary HCMV IgG level (dependent variable; assuming a log-normal distribution) examined the adjusted relations between salivary and serum HCMV IgG levels controlling for all significant oral immune-related and saliva-specific covariates. To assess whether the level of blood or the concentration of total IgG in saliva moderated the adjusted serum-saliva relation for HCMV IgG, we included, in separate models, interaction terms between serum HCMV IgG and salivary transferrin levels and between serum HCMV IgG and salivary total IgG levels.

#### Do Salivary HCMV IgG Test Results Accurately Differentiate HCMV Serostatus Subgroups?

We calculated the sensitivity, specificity, and positive and negative predictive values of the HCMV IgG test in saliva vs. serum using the cut-off values for HCMV antibody levels included in the testing kit. Standard formulae, adjusted to account for equivocal saliva test results, were used for these indices (37). Sensitivity, or the proportion of cases with a positive serum test result that were correctly classified as positive using the saliva test, was calculated as: *n*_true positives_/(*n*_true positives_ + *n*_false negatives_ + *n*_equivocal saliva tests with positive serum tests_). Specificity, or the proportion of cases with a negative serum test result that were correctly classified as negative using the saliva test, was calculated as: *n*_true negatives_/ (*n*_true negatives_ + *n*_false positives_ + *n*_equivocal saliva tests with negative serum tests_). Positive predictive value, or the proportion of cases with a positive saliva test result that also tested positive in serum, was calculated as: *n*_true positives_/(*n*_true positives_ + *n*_false positives_), and negative predictive value, or the proportion of cases with a negative saliva test result that also tested negative in serum, was calculated as: *n*_true negatives_/(*n*_true negatives_ + *n*_false negatives_).

## Results

One participant, with especially high concentrations of all salivary analytes except salivary HCMV IgG (in the top 20th percentile for total IgG, transferrin, IL-1β, IL-6, IL-8, TNFα, and MMP-8), altered the fit of each statistical model conducted and the trends of the observed associations. This participant was excluded from the analytic sample for all analyses (*N* = 98). All linear regression results are presented on the log scale.

### What Are the Distributions and Demographic and Health Correlates of HCMV IgG Levels in Serum and Saliva? Is Salivary HCMV IgG Level Correlated With Serum HCMV IgG Level?

The study sample was majority white and reported overall good physical and oral health (Table 1). Except for race, none of the demographic nor physical health characteristics were significantly associated with HCMV IgG level in serum nor saliva, and they did not vary by HCMV serostatus. Racial homogeneity in the study sample only allowed for statistical comparisons between white (*n* = 44) and African American participants (*n* = 18). On average, African American participants had higher HCMV IgG levels in both serum [*M*_African American_(SD) = 18.44 U/mL (17.12) vs. *M*_white_(SD) = 6.53 U/mL (12.28), *z*(59) = −2.58, *p* < 0.01] and saliva [*M*_African American_(SD) = 8.30 U/mL (7.59) vs. M_white_(SD) = 4.31 U/mL (5.74), *z*(60) = −2.82, *p* < 0.01] compared to white participants. A greater proportion of African American participants were seropositive for HCMV than white participants [56% vs. 16%, respectively, χ^2^(1) = 10.09, *p* < 0.01].

**FIGURE 2:**
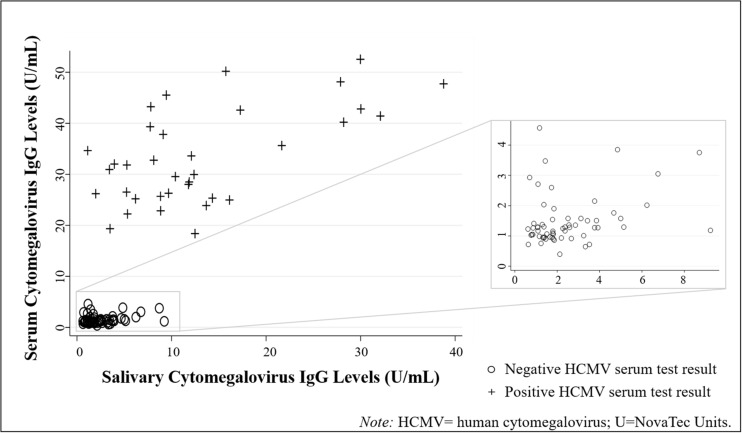
Human cytomegalovirus (HCMV) IgG levels were positively correlated across biospecimen type (*N* = 96 healthy adults). (Data are coded by HCMV serostatus).

Descriptive statistics for all biomeasures are shown in Table 2. HCMV IgG levels were significantly positively correlated across biospecimen [τ(94) = 0.51, *p* < 0.001; Figure 2]. While the serum-saliva relation was numerically stronger among seropositive [τ(33) = 0.29, *p* < 0.05] compared to seronegative participants [τ(59) = 0.15, *p* = 0.08], the unadjusted association between salivary and serum HCMV IgG levels was significant in linear regression models [*b* = 0.05, *t*(94) = 12.05, *p* < 0.001; η^2^ = 0.61], and serostatus did not significantly moderate this association.

**TABLE 2:** Descriptive statistics of biomeasures from a sample of healthy adults.

	Mean	Median	*SD*	Minimum	Maximum	*n*
Serum HCMV IgG (U/mL)^a^	13.09	1.67	16.37	0.39	52.55	96
Salivary HCMV IgG (U/mL)	6.32	3.29	7.78	0.62	38.78	98
Salivary IL-1β (pg/mL)	264.74	209.50	257.26	9.10	1,591.18	98
Salivary IL-6 (pg/mL)	9.79	4.07	16.57	0.35	120.93	98
Salivary IL-8 (pg/mL)	947.70	680.51	743.02	109.11	3,374.14	98
Salivary TNFα (pg/mL)	5.21	2.49	12.04	0.16	110.67	98
Salivary MMP-8 (pg/mL)	76,510.08	56,987.81	57,138.03	5,380.34	200,000.00	92
Salivary Transferrin (mg/dL)	0.69	0.37	0.79	0.04	4.69	98
Salivary Total IgG (μg/mL)	15.40	9.85	16.07	0.40	92.60	98
Salivary Total Protein (mg/mL)	702.99	616.22	367.25	148.53	1,911.19	98

*HCMV, human cytomegalovirus; U, NovaTec Units; IgG, immunoglobulin-G; IL, interleukin; TNFα, tumor necrosis factor-α; MMP-8, matrix metalloproteinase-8; SD, standard deviation. MMP-8 determinations that exceeded the assay’s upper limit of sensitivity (n = 10), were replaced with the upper limit (200,000 pg/mL). Six MMP-8 determinations were unreliable (coefficients of variation >15%) and therefore excluded from analysis. ^a^HCMV IgG levels are biofluid specific, and, given the differences in assay protocols employed, levels in serum and saliva should not be directly compared.*

### Are Salivary HCMV IgG Levels Associated With Oral Immune-Related and Saliva-Specific Confounds?

Salivary HCMV IgG level was significantly positively associated with all biologic measures related to oral inflammation, tissue integrity, and blood leakage into saliva (Table 3). Self-reported oral health composite scores, however, were not significantly associated with salivary HCMV IgG levels. Salivary total protein was also not significantly related to salivary HCMV IgG level.

**TABLE 3:** Salivary human cytomegalovirus IgG levels were positively correlated with all markers related to oral inflammation and tissue integrity (*N* = 98).

	Kendall’s τ correlation coefficient
Salivary IL-1β	0.23***
Salivary IL-6	0.27***
Salivary IL-8	0.19**
Salivary TNFα	0.17*
Salivary MMP-8^a^	0.21**
Salivary Transferrin	0.28***
Oral Inflammation Composite Score	0.23**

IL, interleukin; TNFα, tumor necrosis factor-α; MMP-8, matrix metalloproteinase-8; salivary oral inflammation composite score = standardized mean of salivary IL-1β, IL-6, IL-8, and TNFα. ^a^MMP-8 correlation test n = 92 due to the exclusion of six unreliable determinations with coefficients of variation >15%. MMP-8 relation with salivary HCMV IgG was not statistically significant when cases with determinations above the assay’s limit of sensitivity were excluded from analysis (10 additional cases excluded due to the limits of the assay). *p < 0.05, **p < 0.01, ***p < 0.001.

### Does the Relation Between Salivary and Serum HCMV IgG Levels Strengthen After Accounting for Oral Immune-Related Markers and Salivary Flow Rate? Does the Relation Between Salivary and Serum HCMV IgG Levels Increase With Increasing Levels of Blood and Total IgG in Saliva?

The positive relation between salivary and serum HCMV IgG levels remained significant after adjusting for salivary transferrin, MMP-8, and the oral inflammation composite score [*b* = 0.05, *t*(85) = 15.41, *p* < 0.001; η^2^ = 0.74]. Neither salivary transferrin nor total IgG moderated the serum-saliva association for HCMV IgG.

### Do Salivary HCMV IgG Test Results Accurately Differentiate HCMV Serostatus Subgroups?

Table 4 shows the serum and salivary HCMV IgG test results. Using the serum test result as the indicator of infection status, the saliva test showed a sensitivity of 51% and a specificity of 97%. Every participant with a positive salivary HCMV IgG test also had a positive serum test (100% positive predictive value), and 87% of participants with a negative salivary HCMV IgG test also had a negative serum test (87% negative predictive value).

**TABLE 4:**
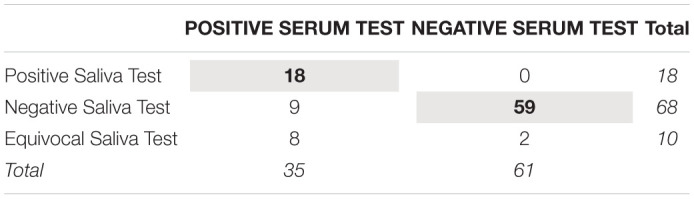
Correspondence between human cytomegalovirus IgG test results using serum and salivary biospecimens from healthy adults (*N* = 96).

*Thresholds for positive, negative, and equivocal test results were defined by the assay kit. Correctly classified cases (saliva test result = serum test result) are shown in gray cells with bolded text.*

### Adjustment for Race, Stratification by Serostatus, and Sensitivity Analyses

Race (white vs. African American) was tested as a covariate in all regression models presented above (subsampled for white and African American participants *n* = 62), and the results showed no significant effects of race in these models and minimal changes in the associations of interest.

Results from models stratified by HCMV IgG serostatus (seropositive *n* = 35; seronegative *n* = 61) were similar to those reported above. As seen with the correlation analyses, in stratified linear regression models, the serum-saliva relation for HCMV IgG was statistically significant among seropositive participants [unadjusted relation: *b* = 0.04, *t*(33) = 3.21, *p* < 0.01; adjusted relation: *b* = 0.04, *t*(27) = 5.44, *p* < 0.001] and not significant or marginal among seronegative participants [unadjusted relation: *b* = 0.15, *t*(59) = 1.53, *p* = 0.13; adjusted relation: *b* = 0.16, *t*(53) = 1.81, *p* = 0.08]. Also, similar to results from models with the full analytic sample, the serum-saliva relation for HCMV IgG was not significantly moderated by salivary total IgG nor transferrin in the seropositive nor seronegative subgroups.

The main findings were robust to influential points and cases with high residuals. However, it is notable that in a sensitivity analysis that excluded one influential case, there was a marginally significant moderating effect of salivary total IgG on the HCMV IgG serum-saliva relation [*b* = −0.00, *t*(82) = −1.84, *p* = 0.07].

## Discussion

Human cytomegalovirus IgG levels measured in whole saliva have the potential to be useful proxies for serum levels. The associations between salivary and serum HCMV IgG levels were robust. That is, the cross-specimen association was strong even after taking into account a range of potential confounders related to the oral environment (e.g., blood contamination and oral inflammation) and participant characteristics. With the exception of race, which showed the expected distribution of higher HCMV antibody levels among African American compared to white participants (38), none of the demographic nor physical health characteristics examined were associated with HCMV antibody levels in serum nor saliva in our sample of healthy adults. Across all participants, for every one unit increase in serum HCMV IgG level, there was approximately a 1.05 unit increase in median salivary levels (median increase approximates increase in the geometric mean after log transformation), and serum HCMV IgG level explained over 60% of the variance in salivary HCMV IgG level (η^2^s > 0.60). This relation was significant in the full analytic sample, and, despite weaker associations among the seronegative compared to the seropositive participants, there was not a statistically significant difference in the magnitude of the serum-saliva association across serostatus groups.

As expected, salivary HCMV IgG was associated with key salivary markers related to oral inflammation (IL-1β, IL-6, IL-8, and TNFα) and tissue integrity (MMP-8). This finding raises a couple interesting possibilities. First, it is well established that these indices reflect inflammatory processes in the local immune and/or mucosal compartment (39). Therefore, it is possible that salivary IgG levels reflect local immunity and viral activity in the oral mucosa and salivary glands, a site of HCMV replication and persistent infection (3, 40). Importantly, the strong serum-saliva association for HCMV IgG levels and the finding that the strength of this association remains when controlling for these inflammatory and tissue integrity related markers strongly suggests otherwise. Given that oral inflammation and tissue damage represent both local immune activation as well as increased permeability of the barriers separating the systemic and local immune systems, our findings of robust serum-saliva relations that hold when the effects of local inflammation are accounted for support the potential utility of a salivary HCMV IgG test in generally healthy individuals without significant oral health problems.

Most of the HCMV IgG measured in saliva is thought to be derived from serum (41), and we anticipated stronger serum-saliva associations for HCMV IgG levels with increasing concentrations of blood in the oral compartment. Interrogation of these relations revealed that, while salivary HCMV IgG was positively related to salivary transferrin, there was no evidence that the strength of the serum-saliva association for HCMV IgG varied by transferrin level. Our findings further suggest that controlling for levels of blood leakage into the oral compartment may not be necessary when assessing HCMV IgG correspondence across serum and salivary samples. This is a tentative conclusion, however, as participants in this study were generally healthy, and individuals with acute and/or chronic physical health conditions and oral health problems were excluded from participation.

Similarly, we expected that the strength of the serum-saliva association for HCMV IgG would vary by the level of total IgG present in the saliva sample. Specifically, we expected cross-specimen associations would be poor without a sufficient level of salivary total IgG. This hypothesis was based on the notion that assay performance would be compromised if the amount of total IgG present in the saliva sample was very low. In this study, the level of salivary total IgG had no significant effect on the serum-saliva association for HCMV IgG. This finding was the same when examined in the full sample, as well as within serostatus subgroups. Results from sensitivity analyses, however, suggest that additional research with larger, more diverse study samples may be required to test these relations.

Despite a robust serum-saliva association for HCMV IgG levels, only about half of the participants who tested positive for HCMV IgG in serum also tested positive in saliva (51% sensitivity). The poor performance of the salivary test in identifying seropositive participants could be due to several factors, most of which may relate to the suboptimal performance of this particular assay when used with saliva. While the findings from our linear regression models showed no evidence of salivary total IgG levels moderating the serum-saliva relation, an indicator of assay performance, we suspect some false negative and equivocal test results from the salivary assay may be related to lower levels of salivary total IgG in the sample. For saliva samples with low total IgG concentrations, assay performance must be very high to capture HCMV antibodies and correctly identify a positive serostatus individual. In these cases, the percent of HCMV antibody in circulation entering the oral cavity may be lower relative to total IgG present in saliva. However, when total IgG levels in saliva are high, it may be easier to identify positive cases. Indeed, findings from our sensitivity analyses suggest total IgG may be playing a role in assay performance, and participants who were misclassified by the salivary test as either negative for HCMV (*n* = 9) or equivocal (*n* = 8) had, on average, lower levels salivary total IgG than true positive cases (i.e., positive test results from both serum and salivary tests (*n* = 18); salivary total IgG- *M*_misclassified_(*SD*) = 10.45 μg/mL (11.26) vs. *M*_true positives_(*SD*) = 29.87 μg/mL (23.83); *z*(33) = 3.17, *p* < 0.01). These misclassified cases, however, also had lower concentrations of salivary transferrin, proinflammatory cytokines, and MMP-8 (*p*’s < 0.05).

With our data, we are not able to delineate key variables underlying the salivary test sensitivity. The specificity of the salivary test, however, was high with nearly all the participants testing negative for HCMV IgG in serum also testing negative in saliva (97% specificity). Furthermore, there were no false positive salivary test results (100% positive predictive value for the salivary test). Future research should examine whether a salivary total IgG threshold could be used in salivary HCMV IgG screening to help decrease the false negative rate and improve the sensitivity for determining exposure status. With this testing criterion in place, assay results from saliva samples that do not meet a cut-off level for total IgG concentration would be considered unreliable, and samples would have to be recollected.

Several limitations of our study warrant discussion. First, this preliminary study used a commercially available diagnostic serum/plasma HCMV IgG assay to determine both salivary and serum HCMV antibody levels. The kit has a reported diagnostic specificity of 99.09% and sensitivity of 99.25%, making it an outstanding measure of exposure status when used in serum. The qualitative results (seropositive vs. seronegative) generated from this assay are based on antibody level determinations that are internally consistent within a biofluid, but estimated relative to the assay specifications, making cross-biospecimen comparisons inappropriate. Relative differences between antibody concentrations within a biofluid are estimated by sample absorbance values compared to the assay controls, so the ranking of antibody determinations, and differences among them, are also biofluid-specific. From a research perspective, these data allow us to examine associations between antibody levels in serum and saliva as well as associations between antibody levels and concentrations of other analytes. However, more advanced methods for determining HCMV antibody levels, particularly in saliva, are needed to support clinical investigations and applications of the research questions examined in this study. Adjusting the saliva sample collection and optimizing assay procedures specifically for use with saliva may improve the performance of future assessments of salivary HCMV IgG levels. For example, the assay protocol calls for repeat testing of samples with equivocal results, which, if instituted in the current study, could have helped classify a greater proportion of our salivary test results into a HCMV exposure group. We also used the cut-off concentrations provided in the kit to determine HCMV status using both serum and salivary biospecimens. Since the kit was designed for serum testing, additional research is needed to determine the most appropriate cut-off values for HCMV IgG levels measured in saliva and the factors that may influence these levels. In addition, we tested saliva samples neat to maximize antibody concentrations and assay performance, and future research should further examine changes in sample collection protocols that could improve assay performance, such as testing antibody-rich gingival crevicular fluid (GCF) or oral mucosal transudate (OMT). These forms of oral fluids are distinct from saliva. In contrast to the composition of saliva, both GCF and OMT represent more of an admixture of serum constituents [see (28) for review]. Not surprisingly, in prior studies, markers of viral infection and inflammation correlate with serum more strongly when measured in these admixture-type oral fluids than saliva [e.g., (42)]. Like whole saliva, acquisition of GCF and OMT is minimally invasive and easily repeatable. Therefore, future studies on the measurement of HCMV IgG levels that include GCF and OMT in comparison to saliva are well worthwhile.

In addition, several other, largely serum-based, HCMV testing approaches are currently available, including via cell culture and immunohistochemistry, IgM and IgA antibody detection, and viral DNA testing methods (25). Assessments of HCMV IgG avidity have also been shown to indicate time since HCMV infection and distinguish between primary and previous infection (43). While HCMV IgG testing can provide valuable information about exposure status, it is unclear how much information about time since exposure and viral shedding HCMV IgG tests alone can provide, especially in the absence of repeat testing (44–46). For saliva-based assessments, studies employing repeated measurements and longitudinal designs are also needed to examine the stability of salivary HCMV IgG levels as several factors, such as flow rate, oral injuries, and immune status, may influence IgG concentrations and affect the accuracy of the salivary HCMV IgG tests. Despite these limitations in interpretability, and technical limitations related to assay sensitivity, there are several advantages of saliva-based HCMV testing, including increased feasibility and acceptability in non-clinical settings and minimally invasive assessment of exposure status, that support the development of a salivary HCMV test for advancing research and public health. Future research should focus on optimizing assay performance as well as explore the utility of alternate saliva-based measures of HCMV infection (e.g., other antibodies and indices of viral shedding).

The generalizability and interpretability of the current findings are also constrained by the relative homogeneity of our study sample and the small number of HCMV positive participants. While the pattern of findings within our analytic sample was robust to sensitivity analyses and extreme and/or influential points, we excluded one participant from our analyses because this participant showed an aberrant pattern of associations and highly influenced the trends observed in the rest of the study sample. This participant had high levels of nearly all salivary analytes, and we believe other, unmeasured, characteristics of this individual may be needed to understand the analyte levels and associations observed (e.g., oral or physical health problems). Additional research conducted in larger, more diverse samples with higher proportions of HCMV seropositive individuals, as well as clinical samples, is needed to fully understand the nature of HCMV IgG levels in saliva and the utility of these measurements for biomedical and research purposes.

It is also important to note two limitations regarding measurements in our study. First, the use of salivary total protein as a surrogate marker for flow rate is disputed in the field (47–51), and future research should assess flow rate and salivary HCMV IgG level relations more directly. Also, 10% (*n* = 10) of our participants had concentrations of salivary MMP-8 that exceeded the assay limits of sensitivity. For these individuals, we replaced their MMP-8 concentrations with the maximum measurable concentration for the assay and used sensitivity analyses to examine whether this substitution approach significantly impacted our findings. When used as a covariate, the main findings were largely unchanged, however, the positive relation between salivary HCMV IgG level and MMP-8 was not significant when participants with MMP-8 concentrations exceeding the assay limits were excluded from analysis. Studies conducted among larger, more heterogeneous samples, with greater diversity in oral and physical health conditions, and a greater proportion of HCMV exposed participants are needed to confirm our findings and broaden our understanding of the promise and potential problems related to salivary HCMV IgG testing.

### Conclusion

With additional research to improve assay performance and optimize the type of oral fluid sampled (i.e., saliva, GCF, or OMT), HCMV IgG testing using oral fluids has the potential to enable minimally invasive screening for HCMV exposure on a large-scale. This possibility could have important implications for public health surveillance and research examining the etiology of HCMV-related diseases. Moreover, there may be clinical applications related to helping determine exposure status among at-risk patients, such as women of childbearing age and the immunocompromised. In addition to expanding testing options for determining HCMV exposure status, salivary measurement of HCMV IgG levels could also open up new opportunities to study the effects of HCMV infection on health in general. HCMV IgG levels have been associated with aging and all-cause mortality (7, 13, 14). If confirmed, our findings of significant cross-specimen correlations for HCMV IgG levels would support these investigations by allowing the minimally invasive measurement of HCMV antibody levels. While additional research using larger and more diverse samples is needed, our findings suggest that, with further technical development, HCMV IgG testing with oral fluids may be an easily-measurable and interpretable proxy for serum HCMV IgG levels. Measuring HCMV IgG levels in oral fluid may be a feasible and reliable approach to advance biobehavioral, clinical, and public health research and practice.

## Data Availability Statement

The datasets presented in this article are not readily available. The data are available upon request to the corresponding author and with the permission of the other lead researchers of this project. Requests to access the datasets should be directed to JR, jriis@uci.edu.

## Ethics Statement

All study procedures were reviewed and approved by the Johns Hopkins University Institutional Review Board. Participants provided their written informed consent to participate in this study.

## Author Contributions

JR, SG, CB, and DG contributed to the conceptualization of the study, analytic plan, and data interpretation. JR, HA, OS, and CB conducted the statistical analyses. JR led the manuscript writing. All authors contributed to the article and approved the submitted version.

## Conflict of Interest

In the interest of full disclosure, DG is founder and chief scientific and strategy advisor at Salimetrics LLC and Salivabio LLC, and these relationships are managed by the policies of the committees on conflict of interest at the Johns Hopkins University School of Medicine and the University of California at Irvine. SG is the Chief Scientific Officer at Salimetrics. The remaining authors declare that the research was conducted in the absence of any commercial or financial relationships that could be construed as a potential conflict of interest.
